# Effect of trace elements and nutrients on diabetes and its complications: a Mendelian randomization study

**DOI:** 10.3389/fnut.2024.1439217

**Published:** 2024-08-01

**Authors:** Ming-Jie Jia, Long Chen

**Affiliations:** The Fourth Clinical Medical College of Guangzhou University of Chinese Medicine, Shenzhen, China

**Keywords:** Mendelian randomization, type 1 diabetes mellitus, type 2 diabetes mellitus, diabetes and its complications, trace elements

## Abstract

**Background:**

Multiple clinical studies have observed a close relationship between serum trace elements and nutrients and diabetes and its complications, but it remains unclear whether there is a genetic causal effect between serum trace elements and nutrients and diabetes and its complications.

**Objective:**

This study aims to investigate the causal effects of serum trace elements and nutrients on diabetes and its complications using Mendelian randomization methods.

**Methods:**

The single nucleotide polymorphisms of serum trace elements and vitamins, as exposure factors, were sourced from the published UK Biobank database and public databases of genome-wide association studies. The genome-wide association study data of diabetes and its complications, as outcome events, were sourced from the FinnGen Biobank database. Mendelian randomization methods were employed to explore the causal relationships between 9 trace elements and 6 nutrients and diabetes and its complications. The causal relationships were inferred using inverse variance weighting, MR Egger, weighted median, simple model, and weighted model methods. Sensitivity analyses, including heterogeneity tests, horizontal pleiotropy tests, MR-PRESSO tests, and leave-one-out analysis, were conducted to evaluate the robustness of the study results. Finally, trace elements and nutrients with statistical significance in the IVW method and consistent Beta and OR directions in the five methods were selected as exposure factors with causal relationships with diabetes and its complications. This study also used multivariable Mendelian randomization methods to assess the combined effects of multiple exposure factors on the risk of diabetes and its complications.

**Results:**

Mendelian randomization analysis revealed that selenium was linked to an elevated risk of T2D.Vitamin B6 was correlated with an increased risk of neurological complications in type 2 diabetes. Magnesium exhibited a negative causal relationship with the risk of T1D.Carotene was linked to a higher risk of renal complications in T1D.Vitamin B12 showed a negative causal relationship with renal complications in T1D.Carotene was connected to a higher risk of neurological complications in T1D.Potassium and vitamin B6 exhibited negative causal relationships with neurological complications in T1D.Vitamin E showed a negative causal relationship with peripheral circulation complications in T2D.Multivariable Mendelian randomization analysis suggested that vitamin B6 could independently influence neurological complications in both T1D and T2D, apart from other exposure factors. Vitamin B6 could also independently influence renal complications in T1D.Vitamin E could independently influence peripheral circulation complications in T1D, apart from other exposure factors.

**Conclusion:**

The findings from univariable and multivariable Mendelian randomization studies substantiate the causal relationships between trace elements and nutrients and different subtypes of diabetes and their complications. These findings hold significant clinical implications for developing targeted prevention and treatment strategies for diabetes and its complications.

## 1 Introduction

Diabetes mellitus is a severe metabolic disease with a continually rising incidence rate. It is projected that by 2030, more than 578 million people globally will be affected (1). As the disease progresses, patients may develop various vascular complications, including large vessel diseases (such as cerebrovascular disease, cardiovascular disease, and peripheral vascular disease) and microvascular diseases (such as diabetic nephropathy, retinopathy, and neuropathy) (2). Vascular complications are the leading cause of death among adults aged 20 to 79 with diabetes (2). Diabetic neuropathy (DN) is the most common complication, encompassing distal symmetric polyneuropathy, small-fiber predominant neuropathy, radiculoplexopathy, and mononeuropathy (3). According to the International Diabetes Federation, by 2050, if not effectively managed, half of type 2 diabetes patients will develop some form of neuropathy (4, 5). Research indicates a close association between peripheral arterial disease (PAD) and diabetes, with approximately 20 to 30% of PAD patients having diabetes (6). Diabetic patients are 2 to 4 times more likely to develop PAD than non-diabetic individuals, and the longer the duration of diabetes, the higher the prevalence (7, 8). Moreover, diabetes worsens the severity of PAD, increasing the risk of amputation (9). Globally, 22.27% of diabetes patients have diabetic retinopathy (DR). In 2020, approximately 103 million people had DR, and this number is projected to rise to 165 million by 2045 (10). Almost all type 1 diabetes patients and 60% of type 2 diabetes patients will develop DR within 20 years, resulting in vision impairment and an increased risk of stroke and coronary heart disease (11, 12). Diabetic nephropathy (DN) often leads to end-stage renal disease (ESRD), with approximately 30% of type 1 diabetes patients and 40% of type 2 diabetes patients developing DN (13, 14). DN is a leading cause of ESRD globally and a risk factor for cardiovascular disease, markedly increasing the risk of adverse cardiovascular events, infections, and mortality (15–17). Diabetes and its complications have a profound economic impact on individuals, families, and society, making the exploration of early prevention and intervention methods essential.

The impact of trace elements and vitamins on diabetes and its complications has garnered widespread attention. Studies have shown that the deficiency or excessive intake of certain trace elements is closely related to the occurrence and development of diabetes and its complications. Early studies indicate that the concentrations of iron, chromium, and copper in the adipose tissue of type 2 diabetes mellitus (T2DM) patients are associated with the risk of developing T2DM, while vanadium and zinc may have protective effects (18). Oost et al. (19) found that magnesium deficiency is associated with increased insulin resistance and cardiovascular disease risk in T2DM patients. Cai et al. (20) suggest that low-dose selenium supplementation can regulate hepatic glycolysis and gluconeogenesis, as well as inhibit oxidative stress, thereby beneficially affecting fasting blood glucose (FBG) levels and glucose tolerance, and reducing the risk of diabetes and its complications. Castillo-Otí et al. (21) found that vitamin D deficiency is associated with an increased incidence of diabetic retinopathy and that vitamin D supplementation can improve retinal health in diabetes patients. Xiao et al. (22) found that vitamin D deficiency is significantly associated with a high prevalence of diabetic foot ulcers in T2DM patients, and that vitamin D screening or supplementation may be beneficial for preventing diabetic foot ulcers and improving outcomes in T2DM patients. Nevertheless, the specific causal relationships between trace elements and nutrients and diabetes and its complications still require further research to be confirmed.

Rigorously designed randomized controlled trials are deemed the gold standard for determining causality and effectively managing potential confounding factors. However, due to ethical constraints, limitations in external validity, challenges in double-blind design, interference from internal and external factors (such as personal behavior, lifestyle, environmental factors), insufficient statistical power, and the need for substantial cost and time, the implementation of randomized controlled trials is often restricted. With the increasing number of large-scale genome-wide association studies (GWAS), Mendelian randomization (MR) has emerged as an effective tool for causal inference between different phenotypes (23). MR is a method that integrates epidemiology and genetics, utilizing naturally occurring genetic variations to simulate randomized experiments for causal inference, thereby more accurately estimating the influence of specific factors on outcomes (24, 25). In MR, single nucleotide polymorphisms (SNPs) related to exposure events are used as instrumental variables. Since instrumental variables are independent of other confounding factors, MR can evaluate the causal relationship between previously observed exposures and outcomes, effectively avoiding confounding bias in traditional epidemiological studies (26). Multivariate Mendelian randomization (MVMR) expands upon MR by considering the causal relationships between multiple exposure factors and diseases. This approach allows MVMR studies to evaluate the joint impact of multiple exposure factors on disease risk, as well as the interactions among these exposure factors (27, 28). Against this backdrop, this study utilizes both two-sample MR and MVMR methods to evaluate the influence of nine trace elements and six nutrients on diabetes and its complications. The goal is to gain deeper insights into the effects of dietary nutrients on diabetes and to offer new perspectives for the future application of micronutrients in the prevention and treatment of diabetes and its complications.

## 2 Materials and methods

### 2.1 Research design

**Figure 1:**
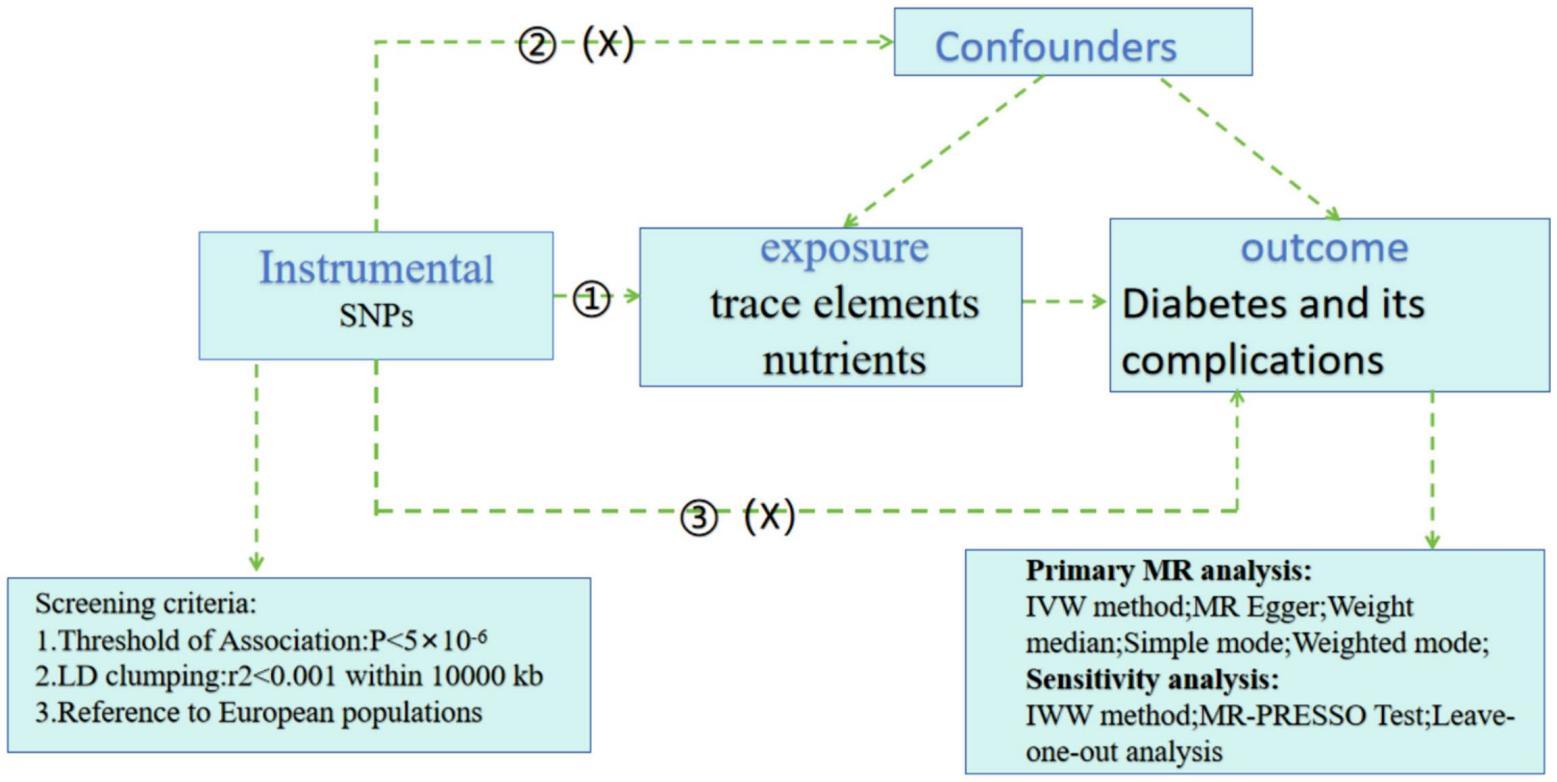
Flow chart of the Mendelian randomization study of trace elements and nutrients on diabetes and its complications.

This study utilizes a two-sample MR study for causal inference, with trace elements (copper, calcium, carotene, folate, iron, magnesium, zinc, potassium, selenium) and nutrients (vitamins A, B12, B6, C, D, and E) as exposure factors, and diabetes and its complications as outcome factors. The three main MR assumptions are illustrated in Figure 1. Assumption 1: The selected SNPs are significantly associated with the exposure factors (trace elements and nutrients). Assumption 2: The SNPs must be independent of potential confounders between the exposure and the outcome. Assumption 3: The SNPs are not directly related to diabetes and its complications, but can only be causally linked through trace elements and nutrients.

### 2.2 Exposure factors and outcome events GWAS data collection

The Genome-Wide Association Studies (GWAS) data for trace elements and nutrients in this study were obtained from IEU OpenGWAS,^1^ and the corresponding GWAS information for trace elements and nutrients are detailed in Table 1. GWAS information for diabetes and its complications was obtained from the Finnish database.^2^ The FinnGen R9 database includes publicly available GWAS data on various diabetes complications. Our study conducted different subtype analyses for type 1 and type 2 diabetes mellitus, covering a range of diabetes complications, including diabetes with renal complications, diabetes with ophthalmic complications, diabetes with neurological complications, and diabetes with peripheral circulatory complications. Diabetes endpoints were defined according to the recommendations of the World Health Organization, and the inclusion and exclusion criteria were based on the International Classification of Diseases,^3^ particularly the 9th or 10th edition (29). Detailed information on the disease data is provided in Table 2.

**Table 1:** GWAS information for trace elements and nutrients.

Trace elements and	GWAS ID	Ancestry	Num_cases	Num_SNPs	Year
Copper	ieu-a-1073	European	2,603	2,543,646	2013
Calcium	ukb-b-8951	European	64,979	9,851,867	2018
Selenium	ieu-a-1077	European	2,603	2,543,617	2013
Zinc	ieu-a-1079	European	2,603	2,543,610	2013
Carotene	ukb-b-16202	European	64,979	9,851,867	2018
Folate	ukb-b-11349	European	64,979	9,851,867	2018
Iron	ukb-b-20447	European	64,979	9,851,867	2018
Magnesium	ukb-b-7372	European	64,979	9,851,867	2018
Potassium	ukb-b-17881	European	64,979	9,851,867	2018
Vitamin A	ukb-b-9596	European	8,863	460,351	2018
Vitamin B12	ukb-b-19524	European	64,979	9,851,867	2018
Vitamin B6	ukb-b-7864	European	64,979	9,851,867	2018
Vitamin C	ukb-b-19390	European	64,979	9,851,867	2018
Vitamin D	ukb-b-18593	European	64,979	9,851,867	2018
Vitamin E	ukb-b-6888	European	64,979	9,851,867	2018

**Table 2:** GWAS information for diabetes and its complications.

Disease categories	Phenocode	Ancestry	num_cases	num_controls
Type 2 diabetes, definitions combined	T2D	European	65,085	335,112
T2D with renal complications	finngen_R9_E4_DM2REN	European	2,684	308,280
T2D with ophthalmic complications	finngen_R9_E4_DM2OPTH	European	4,172	308,280
T2D with neurological complications	finngen_R9_E4_DM2NEU	European	1894	308,280
T2D with peripheral circulatory complications	finngen_R9_E4_DM2PERIPH	European	2,179	308,280
Type1 diabetes, definitions combined	T1D	European	4,320	335,112
T1D with renal complications	finngen_R9_E4_DM1REN	European	1,579	308,280
T1D with ophthalmic complications	finngen_R9_E4_DM1OPTH	European	5,202	308,280
T1D with neurological complications	finngen_R9_E4_DM1NEU	European	1,077	308,280
T1D with peripheral circulatory complications	finngen_R9_E4_DM1PERIPH	European	669	308,280

### 2.3 Instrumental variables

According to the STROBE-MR research guidelines (26), each SNP of trace elements and nutrients undergoes the following screening steps: (i) A genome-wide significance threshold of *p* < 5 × 10^-8 is used, and if the number of significant SNPs under this criterion is small, a threshold of p < 5 × 10^-6 is adopted. (ii) Linkage disequilibrium (LD) testing is performed using the Clump function with criteria set to r^2 < 0.001 and kb = 10,000. (iii) The PhenoScanner database^4^ is used to exclude SNPs associated with outcomes, eliminating confounders. (iv) The F-statistic for each SNP is calculated, and SNPs with *F* < 10 are excluded to avoid bias from weak instrumental variables. Meanwhile, the proportion of exposure explained by the instrumental variable (R^2) is calculated to quantify the strength of the genetic instrument, using the following formula: R^2 = [2 × Beta^2 × (1-EAF) × EAF] / [2 × Beta^2 × (1-EAF) × EAF + 2 × SE^2 × N×(1-EAF) × EAF], where Beta represents the genetic effect of each SNP, EAF is the effect allele frequency, SE is the standard error, and N is the sample size. To evaluate the strength of the selected SNPs, the F-statistic for each SNP is calculated using the following formula: F = R^2(N-k-1) / k(1-R^2), where R^2 represents the proportion of exposure explained by the selected SNPs, N is the sample size, and k is the number of included instrumental variables. SNPs with *F* < 10 are excluded as weak instrumental variables. The remaining independent instrumental variables are used for the subsequent MR analysis (5). MR-PRESSO is used to detect outliers and adjust for horizontal pleiotropy. If horizontal pleiotropy is detected among the instrumental variables, the outliers are removed.

### 2.4 MR analysis

Each trace element and nutrient was assessed for its causal relationship with diabetes and its complications. The potential causal effects were evaluated using the inverse variance-weighted method, weighted median method, weighted mode method, simple mode method, and MR-Egger method. The causal effect reflects the impact of a one standard deviation (SD) increase in each input trace element and nutrient on the risk of outcome characteristics, presented as odds ratio (OR) values and their 95% confidence intervals (CI). Sensitivity analyses included heterogeneity tests, gene pleiotropy tests, and the leave-one-out method. Heterogeneity was tested using Cochran’s Q, where *p* > 0.05 indicates no heterogeneity and *p* < 0.05 suggests possible inter-gene heterogeneity. The ideal outcome of the leave-one-out method is that the results does not change significantly after removing each SNP one by one. Horizontal pleiotropy was detected using the MR-Egger method (commonly expressed by the intercept of MR-Egger) and the MR-PRESSO global test. The above statistical analyses were mainly performed using the TwoSampleMR package (version 0.5.5) of R software (version 4.0.2). All analyses were conducted using the R packages ‘TwoSampleMR (version 0.5.6)’ and ‘MR-PRESSO (version 1.0)’ in R software (version 4.3.1). If the results show no pleiotropy and heterogeneity, IVW is significant, other methods are significant, and the results are stable.

Finally, this study utilized multivariate Mendelian randomization research to analyze the causal relationships between multiple exposure factors and disease, evaluating the joint impact of multiple exposure factors on disease risk and their interactions. Through these analyses, this study aims to deepen the understanding of the causal relationships between trace elements and vitamins and diabetes and its complications, and to provide scientific evidence for the prevention and treatment of the disease.

## 3 Result

### 3.1 Instrumental variables screening results

SNPs meeting the three main assumptions were selected based on the set criteria, and variables that might affect the outcomes were excluded using the PhenoScanner database. The *F*-values of the remaining instrumental variables were all greater than 10. When using the genome-wide significance threshold of *p* < 5 × 10^-8, the number of available SNPs was too small to analyze. Therefore, according to the STROBE-MR guidelines and literature review, the *p*-value was set to p < 5 × 10^-6. Finally, 188 SNPs significantly associated with all trace elements were identified as instrumental variables. Detailed information on the instrumental variables can be found in the Supplementary material.

### 3.2 Mendelian randomization results

**Figure 2:**
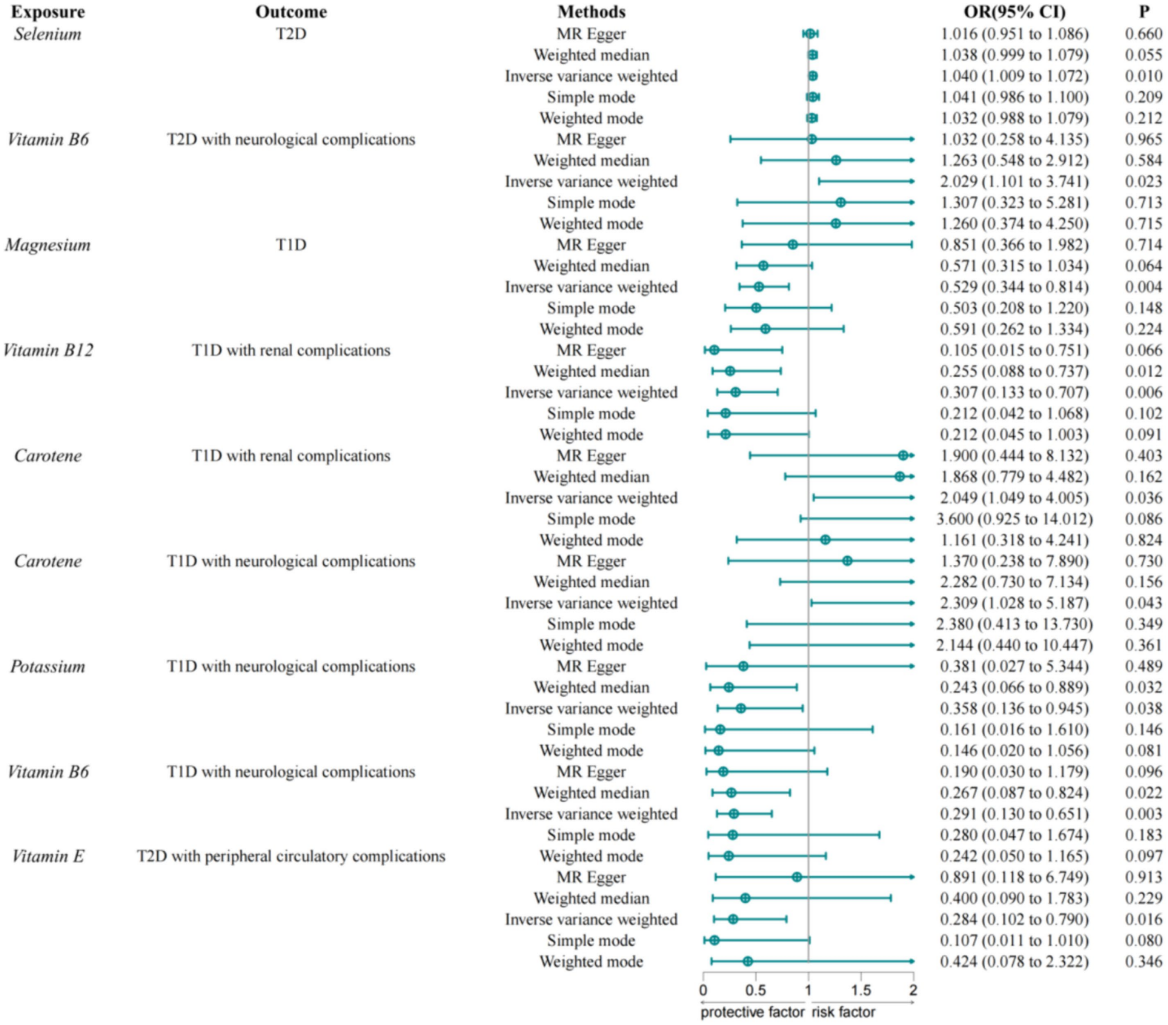
MR results of trace elements and nutrients and diabetes and its complications.

Using the IVW method as the primary result, we found that the selenium is associated with an increased risk of type 2 diabetes (OR = 1.040, 95% CI = 1.009–1.072, *p* = 0.010) (Figure 2). Vitamin B6 is associated with an increased risk of type 2 diabetes with neurological complications (OR = 2.029, 95% CI = 1.101–3.741, *p* = 0.023). The study found no effect of trace elements and nutrients on type 2 diabetes with ophthalmic complications, peripheral circulatory complications, and renal complications. Magnesium is negatively causally related to the risk of type 1 diabetes (OR = 0.529, 95% CI = 0.344–0.814, *p* = 0.004), suggesting that magnesium may be a protective factor against type 1 diabetes. Carotene is associated with an increased risk of type 1 diabetes with renal complications (OR = 2.049, 95% CI = 1.049–4.005, *p* = 0.036). Vitamin B12 is negatively causally related to type 1 diabetes with renal complications (OR = 0.307, 95% CI = 0.133–0.707, *p* = 0.006). Carotene is associated with an increased risk of type 1 diabetes with neurological complications (OR = 2.309, 95% CI = 1.028–5.187, *p* = 0.043). Potassium (OR = 0.358, 95% CI = 0.136–0.945, *p* = 0.038) and vitamin B6 (OR = 0.291, 95% CI = 0.130–0.651, *p* = 0.291) are negatively causally related to type 1 diabetes with neurological complications. Vitamin E is negatively causally related to type 2 diabetes with peripheral circulatory complications (OR = 0.284, 95% CI = 0.102–0.790, *p* = 0.016). No effect of trace elements and vitamins on type 1 diabetes with ophthalmic complications was found. To avoid excessive bias, we conducted a series of sensitivity analyses to test the reliability of MR analyses and to detect potential horizontal pleiotropy. The intercept of the MR-Egger showed no horizontal pleiotropy for all causal effects (*p* > 0.05). Cochran’s Q and leave-one-out tests indicated no significant heterogeneity. This suggests that the MR analysis results are robust.

### 3.3 Results of the multivariate Mendelian study

Through multivariable Mendelian randomization studies, we analyzed the causal relationships between multiple exposures and diseases, assessed the combined impact of multiple exposures on disease risk, and evaluated the interactions among these exposures.

**Figure 3:**
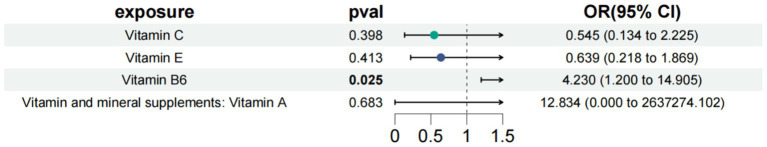
Results of a multivariate Mendelian study of type 2 diabetes with neurological complications.

In the study of type 2 diabetes with neurological complications, we used vitamin B6, vitamin C, vitamin E, and vitamin A as the exposure factors to assess the combined impact of these exposure factors on disease risk and their interactions. The results showed that under the combined effect of multiple vitamins, vitamin B6 still had a direct impact on type 2 diabetes with neurological complications (OR = 4.230; 95% CI =1.200–14.905; *p* = 0.025) (Figure 3). Sensitivity analysis showed no horizontal pleiotropy or heterogeneity for all causal effects, indicating robust MR analysis results. This suggests that vitamin B6 can independently affect type 2 diabetes with neurological complications, separate from vitamins C, E, and A.

**Figure 4:**
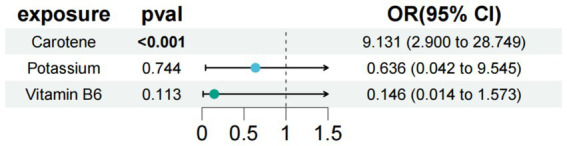
Results of a multivariate Mendelian study of type 1 diabetes with neurological complications.

In the study of type 1 diabetes with neurological complications, we used carotene, potassium, and vitamin B6 as exposure factors to assess the combined impact of these exposure factors on disease risk and their interactions. The results showed that under the combined effect of multiple exposure factors, carotene still had a direct impact on type 1 diabetes with neurological complications (OR = 9.131; 95% CI =2.900–28.749; *p* < 0.001) (Figure 4). Sensitivity analysis showed no horizontal pleiotropy or heterogeneity for all causal effects, indicating robust MR analysis results. This suggests that carotene can independently affect type 1 diabetes with neurological complications, separate from potassium and carotene.

**Figure 5:**
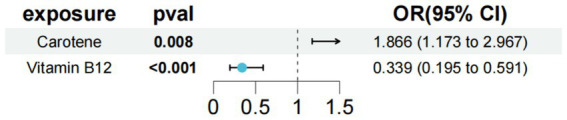
Results of a multivariate Mendelian study of type 1 diabetes with peripheral circulatory complications.

In the study of type 1 diabetes with renal complications, we used carotene and vitamin B12 as exposure factors to assess the combined impact of these exposure factors on disease risk and their interactions. The results showed that under the combined effect of multiple exposure factors, vitamin B12 still had a direct impact on type 1 diabetes with renal complications (OR = 0.339; 95% CI =0.195–0.591; *p* < 0.001) (Figure 5). Sensitivity analysis showed no horizontal pleiotropy or heterogeneity for all causal effects, indicating robust MR analysis results. This suggests that vitamin B12 can independently affect type 1 diabetes with renal complications, separate from carotene.

**Figure 6:**
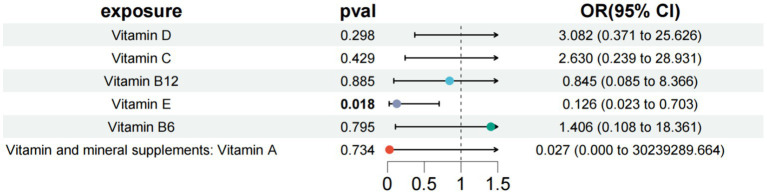
Results of a multivariate Mendelian study of type 1 diabetes with peripheral circulatory complications.

In the study of type 1 diabetes with peripheral circulatory complications, we used vitamin D, vitamin C, vitamin B12, vitamin E, and vitamin B6 as exposure factors to assess the combined impact of these exposure factors on disease risk and their interactions. The results showed that under the combined effect of multiple vitamins, vitamin E still had a direct impact on type 1 diabetes with peripheral circulatory complications (OR = 0.126; 95% CI =0.023–0.703; *p* = 0.018) (Figure 6). Sensitivity analysis showed no horizontal pleiotropy or heterogeneity for all causal effects, indicating robust MR analysis results. This suggests that vitamin E can independently affect type 1 diabetes with peripheral circulatory complications, separate from carotene.

## 4 Discussion

To our knowledge, this study is the first to evaluate the effects of trace elements and vitamins on different subtypes of diabetes and its complications using Mendelian randomization (MR) methods. The study used published GWAS data to infer the causal relationship between serum trace elements and vitamins and different subtypes of diabetes and its complications. The analysis results revealed that selenium is associated with an increased risk of developing type 2 diabetes. Vitamin B6 is associated with an increased risk of neurological complications in type 2 diabetes. Magnesium is negatively related to the risk of developing type 1 diabetes. Carotene is associated with an increased risk of kidney complications in type 1 diabetes. Vitamin B12 is negatively related to the risk of kidney complications in type 1 diabetes. Carotene is associated with an increased risk of neurological complications in type 1 diabetes. Potassium and vitamin B6 are negatively related to the risk of neurological complications in type 1 diabetes. Vitamin E is negatively associated with the risk of peripheral circulatory complications in type 2 diabetes. The multivariable Mendelian randomization analysis results showed that Vitamin B6 can affect type 1 diabetes with neurological complications and type 2 diabetes with neurological complications independently of other exposure factors. Vitamin B6 may affect type 1 diabetes with renal complications independently of other exposure factors. Vitamin E may affect type 1 diabetes with peripheral circulatory complications independently of other exposure factors.

Selenium is an essential trace element in the human body, garnering significant attention due to its dual role in health and disease. However, the relationship between selenium and diabetes is complex and appears to be dose-dependent. Research has shown that elevated serum selenium levels are related to an increased risk of type 2 diabetes (T2D), aligning with our results. A randomized controlled trial discovered that supplementing 200 μg of selenium daily might increase the risk of type 2 diabetes (30). Another secondary analysis of a nasopharyngeal cancer trial suggested that during 7.7 years of follow-up, individuals (mostly men) who achieved relatively high serum selenium levels (around 190 μg/L) by daily supplementation with 200 μg of selenium yeast showed an increased incidence of type 2 diabetes (31). Multiple cross-sectional and cohort studies jointly found that serum selenium levels between 124 and 147 μg/L are positively associated with hyperglycemia and self-reported prevalence of T2D (32–35). Research suggests that selenoprotein P (SELENOP) produced by the liver can transport selenium to peripheral tissues, and that elevated serum SELENOP levels may result in decreased insulin sensitivity and impaired glucose tolerance (36, 37). Excessive selenium may lead to oxidative stress, harming pancreatic β-cells and insulin-sensitive tissues, eventually increasing the risk of type 2 diabetes (38, 39). Furthermore, glutathione peroxidase 1 (GPX1), a selenium-containing antioxidant enzyme, has been discovered to inhibit insulin signaling by lowering reactive oxygen species levels, resulting in insulin resistance and possibly affecting pancreatic β-cell function (38, 39). Huang et al. (40) noted that an increase in selenium primarily affects oxidative stress, insulin signalling, selenium metabolism, and the expression of specific selenoproteins, thereby increasing the risk of type 2 diabetes. Selenoprotein P (SELENOP) is a selenium-containing protein that has been found to impact insulin signalling and glucose metabolism negatively. Elevated levels of SELENOP are associated with decreased insulin sensitivity and an increased risk of type 2 diabetes. Experimental studies have demonstrated that SELENOP affects the phosphorylation of key molecules in the insulin signaling pathway, thereby impairing insulin signaling and leading to insulin resistance in hepatocytes and myocytes (41). Over time, this disruption can result in reduced insulin sensitivity and elevated blood glucose levels, heightening the risk of developing diabetes. Furthermore, meta-analyses have confirmed the association between high circulating levels of SELENOP and various glucose and lipid metabolism disorders. These conditions include gestational diabetes, metabolic syndrome, non-alcoholic fatty liver disease, obesity, and type 2 diabetes (42). Given its close relationship with markers of metabolic dysfunction, SELENOP can serve as a biomarker for these diseases. Nevertheless, these mechanisms need further investigation to be fully elucidated.

Research indicates that elevated levels of vitamin B6 are linked to an increased risk of neurological complications in type 2 diabetes, consistent with our findings (43). Although vitamin B6 is beneficial at regular doses, high-dose supplementation may induce neurotoxicity, resulting in sensory axonal neuropathy. This neuropathy usually alleviates after stopping high-dose vitamin B6 intake (44). Vitamin B6 is inversely correlated with homocysteine levels, and diabetic patients with peripheral neuropathy often exhibit reduced vitamin B6 levels. Vitamin B6 is a crucial coenzyme in the metabolism of homocysteine, participating in the transsulfuration pathway. In type 1 diabetes patients, due to insufficient or absent insulin secretion, blood glucose levels are chronically elevated. This chronic hyperglycemic condition may influence the metabolism of homocysteine. If vitamin B6 levels are inadequate, it may result in homocysteine metabolism disorders, thereby impacting nervous system health. Additionally, vitamin B6 deficiency may affect the nitric oxide neurotransmitter system, potentially reducing NMDA receptor function, thereby impacting nitric oxide synthase activity, leading to decreased nitric oxide synthesis and further promoting the development of diabetic peripheral neuropathy (45–47). Research indicates that both vitamin B6 deficiency and elevated levels may be related to peripheral neuropathy, with high levels potentially worsening diabetes-related neurological issues (45). In summary, although vitamin B6 is vital for human health, its intake should be cautious, particularly for type 2 diabetes patients, as excessive supplementation may cause adverse neurological effects. For type 1 diabetes patients, vitamin B6 supplementation might have a protective effect; however, its specific impact and mechanisms require further study. Additionally, for diabetic patients requiring vitamin B6 supplementation, physicians should balance the benefits and risks and choose the appropriate dose and form to minimize the risk of neurotoxicity.

Numerous studies (48, 49) indicate that type 1 diabetes patients commonly exhibit decreased serum magnesium levels, particularly among children and adolescents. Low serum magnesium levels are closely related to poor glycemic control, increased risk of complications, and early development of cardiovascular complications. Oost et al. (50) found that in type 1 diabetes patients receiving high doses of insulin therapy, hypomagnesemia may exacerbate insulin resistance, thereby affecting glycemic control and inflammation levels. Additionally, research suggests (51) that magnesium supplementation can improve glycemic control and lipid profiles in children with type 1 diabetes, indicating that magnesium supplementation may have a positive effect on metabolic control in type 1 diabetes patients. A systematic review and meta-analysis (52) also support the association between decreased magnesium levels and poor glycemic control and/or complications in type 1 diabetes patients. Moreover, decreased magnesium levels are associated with elevated levels of triglycerides, total cholesterol, and low-density lipoprotein cholesterol, as well as reduced high-density lipoprotein cholesterol levels. These findings further underscore the importance of magnesium in metabolic regulation in type 1 diabetes patients. Additionally, magnesium deficiency may interfere with enzymatic reactions in the insulin signaling pathway or induce inflammation or oxidative stress, thereby affecting glucose metabolism (53). Although this condition is usually observed in type 2 diabetes, specific studies on type 1 diabetes still require further exploration and validation. In summary, abnormalities in magnesium metabolism play a crucial role in type 1 diabetes, affecting disease progression and patients’ metabolic status. Therefore, intervention strategies targeting magnesium metabolism may offer new directions for the treatment of type 1 diabetes. Future research should delve into the mechanisms of magnesium in type 1 diabetes and the potential clinical applications of magnesium supplementation.

Du et al. evaluated the effects of carotenoids from Sporidiobolus pararoseus on STZ-induced diabetic nephropathy (DN) in mice. The results showed (54) that these carotenoids significantly alleviated DN symptoms, including reduced fasting blood glucose, decreased urine output, urinary albumin, serum creatinine, and blood urea nitrogen, and improved renal histological morphology. Additionally, they improved oxidative stress status in DN mice by activating the expression of Nrf2, NQO-1, HO-1, GST, and CAT in the kidneys. Shi et al. found (55) that higher carotenoid intake in women was significantly negatively correlated with the prevalence of chronic kidney disease (CKD). However, our findings showed that carotenoids were associated with an increased risk of type 1 diabetes with renal complications, which is inconsistent with previous conclusions. One possible explanation is the difference in study populations and disease types. Furthermore, we focused on the total intake of carotenoids rather than specific sources of carotenoids. Therefore, further in-depth studies may be needed to explore the effects of carotenoids on diabetic nephropathy.

Wu et al. found (56) that supplementation with folic acid and vitamin B12 can reduce serum homocysteine and malondialdehyde levels in patients with diabetes and diabetic nephropathy, decrease the excretion of 24-h urinary microalbumin, and increase levels of nitric oxide and superoxide dismutase. Additionally, with the progression of kidney disease, vitamin B12 levels in T2DM patients decrease while homocysteine levels increase (57). Studies have shown that vitamin B12 has neuroprotective effects in streptozotocin-induced type 1 diabetic rats (58). Specifically, vitamin B12 can reduce neuron apoptosis induced by type 1 diabetes, improve neurotrophic support and synaptic plasticity, and mitigate astrocyte proliferation and endoplasmic reticulum stress (58). In summary, vitamin B12 supplementation not only helps improve the symptoms and progression of diabetic nephropathy but also shows potential significance in neuroprotection. However, there is currently insufficient evidence to support the positive effects of vitamin B12 on kidney complications in type 1 diabetes, although its neuroprotective role is more prominent.

Potassium ions are crucial for maintaining normal nerve cell function, especially in nerve impulse conduction and muscle contraction. The balance of potassium ions is vital for the health of the nervous system. Studies have shown (59) that potassium ion channels play a key role in neuronal excitability and conduction. Imbalances in potassium ion levels can affect nerve function, leading to nerve conduction disorders and neuropathy (59). Additionally, potassium deficiency may exacerbate symptoms of diabetic neuropathy, such as pain and numbness (59). Research indicates (59, 60) that low potassium levels may worsen symptoms of diabetic neuropathy because the lack of potassium’s key role in nerve conduction can lead to neuronal dysfunction, thereby worsening neuropathy. In conclusion, there is a negative causal relationship between potassium and neurological complications in type 1 diabetes, and maintaining appropriate potassium levels may help alleviate these symptoms.

Research indicates that vitamin E significantly contributes to reducing oxidative stress and enhancing vascular health, which is vital for managing diabetes-related peripheral vascular complications. A study highlighted the protective role of antioxidant nutrients, including vitamin E, stating that vitamin E improves endothelial function and reduces oxidative stress (61). The findings of Bi′s study confirm that vitamin E can substantially decrease the occurrence and severity of peripheral circulatory complications in diabetic patients by mitigating oxidative stress and enhancing vascular health (62). Thus, appropriate vitamin E supplementation could be a viable approach to preventing and treating peripheral vascular complications in diabetes.

Oxidative stress plays a pivotal role in the onset and progression of diabetes and its associated complications. Under hyperglycemic conditions, oxidative stress induces cellular damage and inflammatory responses, subsequently leading to complications such as cardiovascular diseases, retinopathy, and nephropathy (63, 64). Previous studies have reported the impact of trace elements and nutrients on oxidative stress. Selenium, as an essential component of glutathione peroxidase (GPx) and thioredoxin reductase (TrxR), is crucial in eliminating peroxides and protecting cells from oxidative damage (65). However, an excess of selenium can disrupt redox homeostasis, resulting in heightened oxidative stress and insulin resistance, thereby exacerbating the complications of diabetes (38–40). Therefore, strict control of selenium supplementation within a safe range is imperative to harness its beneficial effects while avoiding potential harm. Vitamin C, a water-soluble antioxidant, can directly neutralize free radicals, protecting both intracellular and extracellular molecules from oxidative damage. Additionally, it can regenerate other antioxidants such as vitamin E (64, 66). Given the high oxidative stress levels typically present in diabetic patients, vitamin C supplementation can alleviate oxidative stress, improve insulin sensitivity, and reduce the incidence of cardiovascular complications associated with diabetes (67). Vitamin E, predominantly located in cell membranes, protects membrane integrity by inhibiting lipid peroxidation. It neutralizes lipid radicals, thereby preventing oxidative damage to cell membranes (68). Research indicates that vitamin E supplementation can delay the onset and progression of diabetic complications, enhancing vascular endothelial function, retinal blood flow, and renal function in diabetic patients (69). Moreover, copper and manganese are integral components of superoxide dismutase (SOD). SOD catalyzes the dismutation of superoxide anions into oxygen and hydrogen peroxide, which is subsequently decomposed by other antioxidant enzymes such as catalase (70). Thus, adequate levels of copper and manganese are deemed beneficial for maintaining the normal function of the antioxidant defense system, mitigating oxidative stress-related damage in diabetes, and reducing the risk of complications. In summary, the relationship between trace elements, nutrients, and oxidative stress is complex and interrelated. Further research is warranted to comprehensively understand the role of trace elements and nutrients in the pathogenesis and progression of diabetes and its complications through oxidative stress mechanisms.

Diet is the primary source of trace elements and nutrients, and different dietary patterns have a significant impact on the nutritional status of the body. High-carbohydrate diets are typically energy-dense but may be deficient in trace elements and vitamins, particularly those crucial for glucose metabolism and antioxidant defense, such as chromium, magnesium, and the B vitamins. Conversely, diets high in meat products may provide higher intakes of iron and zinc (71). Diets with high intakes of fruits and vegetables are usually associated with higher intakes of vitamin C, vitamin E, β-carotene, and selenium, which are essential for protecting cells from oxidative damage (72, 73). Fruits and vegetables are also rich in fiber, which is vital for maintaining gut health and stabilizing blood glucose levels. Diets low in fruit and vegetable intake may lead to deficiencies in these key nutrients, increasing the risk of diabetes and other chronic diseases. Epidemiological studies have shown an association between dietary patterns and the risk of developing diabetes. High-carbohydrate diets, especially those rich in refined sugars and refined grains, are linked to an increased risk of type 2 diabetes (74, 75). This may be due to the rapid fluctuations in blood glucose and insulin levels caused by high-carbohydrate diets, as well as the decreased antioxidant defense capacity related to inadequate intake of trace elements and vitamins. In contrast, the relationship between high meat consumption and diabetes risk is more complex. Studies have indicated that high red meat intake is associated with an increased risk of diabetes, while high intake of white meat and fish is associated with a lower risk (76). This could reflect differences in the fat and trace element content of different types of meat. Diets high in fruit and vegetable intake are generally associated with a lower risk of diabetes (77). This may be attributed to the high levels of antioxidants and fiber in these foods, which help improve blood glucose control and reduce oxidative stress. In summary, dietary patterns significantly influence the intake of trace elements and nutrients, thereby affecting the risk of developing diabetes. A balanced diet rich in whole grains, fruits, and vegetables, along with moderate consumption of meat products, may be an effective strategy for preventing and managing diabetes.

Cardiovascular disease is one of the primary complications of diabetes, with trace elements playing a crucial role in maintaining cardiovascular health. The antioxidant properties of selenium and zinc protect vascular endothelial cells from oxidative damage (78). Endothelial dysfunction is a key factor in atherosclerosis, the primary pathological basis of cardiovascular disease (79). Thus, the antioxidant defense capabilities of selenium and zinc are significant for preventing atherosclerosis and its complications. Elastin and collagen are major components of the vascular wall, with their structure and function directly affecting the elasticity and tensile strength of blood vessels. Copper is essential in the synthesis of these proteins (80). Defects in copper metabolism can lead to cardiac and muscle dysfunction, thereby increasing the risk of heart disease (81). Additionally, copper plays a role in collagen synthesis and antioxidant defense; supplementation can improve endothelial function and reduce oxidative stress (82). Low selenium levels are associated with an increased risk of cardiovascular disease, and selenium supplements can reduce oxidative stress and inflammation (78). Magnesium is vital for protein synthesis, muscle and nerve function, and blood pressure regulation. Low magnesium levels are linked to an increased risk of cardiovascular disease, and magnesium supplementation has been shown to lower blood pressure and improve endothelial function (78). Withaferin A (WA), an active component of *Withania somnifera*, exhibits a diverse range of biological activities including anti-inflammatory, immunomodulatory, anti-stress, antioxidant, and anti-angiogenic properties. WA inhibits cancer cell growth through multiple mechanisms, such as inducing apoptosis, suppressing cell migration and invasion, and regulating the cell cycle, demonstrating significant anti-cancer activity across various cancer types (83). Diabetic patients typically experience elevated levels of oxidative stress, which can lead to cellular damage and inflammatory responses, thereby increasing the risk of cardiovascular disease. The antioxidant and anti-inflammatory properties of WA may help reduce the occurrence of cardiovascular complications. Future research should explore the synergistic effects of micronutrients and WA in the prevention and management of cardiovascular issues in diabetic patients. Furthermore, studies have found that there are differences between women and men in terms of cardiovascular disease risk and pathogenesis (84). Future research should focus more on the relationship between trace elements and cardiovascular health in women. This includes studies on gender differences, intervention measures, and clinical trials, to understand the role of trace elements in women’s cardiovascular health. Such research could provide new insights and methods for the prevention and treatment of cardiovascular diseases in women. In summary, the impact of trace elements on cardiovascular function is multifaceted, encompassing antioxidant properties and the maintenance of vascular structure and function. Considering the role of trace elements in the study of diabetic complications is essential for a comprehensive understanding of disease mechanisms and the development of effective treatment strategies. Therefore, the influence of trace elements on cardiovascular function, including their antioxidant effects and roles in maintaining vascular structure and function, is paramount in understanding and managing diabetic complications.

In 2022, the European Society of Clinical Nutrition and Metabolism (ESPEN) published micronutrient guidelines (85). We have summarized the common micronutrients covered in them for adequate intake, dietary reference intake, enteral nutrition, tolerable upper intake level, parenteral nutrition, recommended dietary allowances, and risk of deficiency and excess, which are summarized in the Table 3. By referring to ESPEN’s micronutrient guidelines, clinicians can be helped to more accurately assess the nutritional needs of patients with diabetes, better formulate nutritional support programmes to ensure that they receive adequate and not excessive amounts of micronutrients, optimize health outcomes, and improve patients’ quality of life.

**Table 3:** Recommended amounts of micronutrients and risks of deficiencies and excesses.

Micronutrient	Recommended intake	Risk of deficiency	Risks of excess
Chromium	Oral AI: 35 μg/day for men, 25 μg/day for women PN:10–15 μg/day	It may be associated with glucose metabolism disorders, weight loss, and neuropathy, particularly in patients with acute illnesses and those receiving long-term parenteral nutrition.	Long-term high-dose supplementation may lead to kidney function impairment.
Copper	Oral DRI: 1.1–2.0 mg/day PN: 0.3–0.5 mg/day	It may cause anemia, neutropenia, osteoporosis, and hair depigmentation. High-risk groups include patients with major burns, post-gastrointestinal surgery patients, and long-term parenteral nutrition patients.	Excess can lead to hepatotoxicity, kidney failure, behavioral changes, and neurotoxicity.
Selenium	Adult RDA: 55 ug/day UL: 400 ug/day	Deficiency can lead to Keshan disease, cardiomyopathy, weakened immune function, and thyroid dysfunction.	Excess can lead to selenium toxicity, with symptoms including hair loss, nail deformities, gastrointestinal discomfort, and neurological symptoms.
Molybdenum	Adult RDA: 45 ug/day UL: 2000 ug/day	Deficiencies are very rare, but can lead to metabolic problems.	Excessive amounts may cause copper deficiency, joint pain and other metabolic problems.
Zinc	Adult RDA: 11 mg/day for men, 8 mg/day for women UL: 40 mg/day	Deficiencies can lead to reduced immune function, skin lesions, loss of appetite and growth retardation.	Excessive intake leads to copper deficiency, reduced immune function and elevated cholesterol levels.
Iron	Adult RDA: 8 mg/day for men, 18 mg/day for women UL: 45 mg/day	Deficiency can lead to anaemia, fatigue and reduced immune function.	Excessive intake may lead to iron toxicity manifested by gastrointestinal distress, liver damage and increased oxidative stress.
Calcium	Adult RDA: 1000 mg/day UL: 2500 mg/day	Deficiencies can lead to osteoporosis, fractures and muscle spasms.	Excessive intake may lead to hypercalcaemia, kidney stones and soft tissue calcification.
Iodine	Adult RDA: 150 ug/day UL: 1100 ug/day	Deficiency can lead to goiter, hypothyroidism and developmental delay.	Excessive intake may lead to hyperthyroidism and goiter.
Fluoride	AI& UL: 10 mg/day	Deficiencies can lead to dental caries and bone health problems.	Excessive intake can lead to fluorosis, which manifests itself as abnormal hardening of bones and teeth.
Cobalt	No clear DRI, ingested mainly as part of vitamin B12	As part of vitamin B12, deficiency can lead to anaemia and neurological problems.	Excessive amounts may cause cardiomyopathy and other signs of toxicity, especially when using metal implants containing cobalt.

AI, adequate intake; DRI, dietary reference intake; EN, enteral nutrition; UL, tolerable upper intake level; PN, parenteral nutrition; RDA, recommended dietary allowances.

The strengths of this study are as follows: First, the analysis results indicate that magnesium, vitamin B12, potassium, vitamin B6, and vitamin E have protective effects against diabetes and its complications, whereas selenium, vitamin B6, and carotene are risk factors for diabetes and its complications. Therefore, for high-risk clinical populations, controlling the intake of selenium and carotene and appropriately supplementing magnesium, potassium, vitamin B12, and vitamin E in daily life may be a potential measure for preventing diabetes and its complications. Additionally, this study provides possible experimental directions for researching the molecular mechanisms through which trace nutrients influence diabetes, laying a research foundation for the future prevention and treatment of diabetes and its complications from the perspective of biological trace nutrients. Second, the study used publicly available GWAS data for causal inference, resulting in a larger sample size and higher statistical power.

However, this study has several limitations: First, the study is limited to European populations, and the results may not be generalizable to populations with different genetic backgrounds. There could be significant differences in various racial backgrounds. Second, this study is primarily a foundational theoretical research and requires more animal experiments and cohort studies to confirm the conclusions before they can be better applied in clinical settings. Additionally, the different impacts of trace elements and nutrients on type 1 and type 2 diabetes and their complications can be attributed to the distinct pathogenic mechanisms of the two types. Type 1 diabetes is caused by the extensive destruction of pancreatic β-cells, ultimately leading to absolute insulin deficiency. Type 2 diabetes primarily stems from insulin resistance and progressive deterioration of insulin secretion. Due to their different pathogenic mechanisms, their effects on different subtypes of diabetes and its complications vary. Furthermore, type 2 diabetes mainly occurs in middle-aged and older adults (typically over the age of 40), whereas type 1 diabetes usually occurs in children and adolescents, who generally have different intakes of trace elements and nutrients. This factor may also play a role in elucidating their differential impacts between the subtypes.

We have found that the intake of micronutrients may have a nonlinear relationship with health outcomes, using selenium as an example. Research has shown that when selenium intake exceeds the nutritionally required levels, the risk of T2D increases with higher selenium intake. However, it is currently uncertain whether low selenium status promotes T2D risk in humans. Although case–control studies have suggested a U-shaped response of T2D risk to selenium status, with approximately 100 μg/L of serum selenium being the point of lowest risk, this has not been supported by cohort or cross-sectional studies. In animal studies, researchers compared different dietary selenium levels and specific selenoproteins in a homogeneous background, finding that low selenium status is associated with increased T2D risk. These studies collectively suggest that increased oxidative stress in both high and low selenium status may be a mechanism leading to higher T2D risk. Overall, current evidence indicates that T2D risk is minimized at the bottom of the U-shaped dose–response curve for selenium intake, meaning that both selenium deficiency and excessive supplementation can increase the risk.

We propose several potential reasons for the nonlinear relationship between micronutrient intake and health outcomes: (i) Optimal Range: Each micronutrient has an optimal intake range within the body, where it can most effectively support health. Intake below this range can lead to deficiency diseases, affecting normal physiological functions, while intake above this range can cause toxicity and health problems (refer to Table 3). (ii) U-shaped or J-shaped Curve: Many micronutrients exhibit U-shaped or J-shaped dose–response relationships, indicating that both insufficient and excessive intake increase the risk of diseases, with moderate intake being the safest (refer to Table 3). (iii) Regulatory Mechanism Limitations: The body has complex mechanisms to regulate micronutrient levels. For example, when the intake of a specific nutrient is low, the body increases its absorption and reduces its excretion. However, when intake is too high, these regulatory mechanisms may fail, leading to increased risk from overconsumption.(iv) Nutrient Interactions: There are interactions between different micronutrients that affect their bioavailability. For instance, high doses of zinc can lead to copper deficiency (refer to Table 3). Therefore, excessive intake of one nutrient can cause deficiency in another, resulting in health problems. (v) Individual Differences: Factors such as genetics, age, sex, and health status can affect the optimal intake levels of micronutrients. For example, the nutritional needs of pregnant and lactating women differ from those of the general adult population, leading to varying risks and impacts of under- or overconsumption.

In summary, the nonlinear relationship between micronutrient intake and health outcomes is primarily due to the body’s complex regulatory mechanisms, bioavailability, individual differences, and nutrient interactions. Therefore, we recommend conducting further large-scale, long-term cohort studies to confirm and refine the nonlinear relationship between micronutrient intake and health outcomes. Additionally, personalized nutritional assessment and supplementation plans should be developed based on individual health status, lifestyle, and genetic background. Regular monitoring of patients’ micronutrient levels is essential, with dynamic adjustments to supplementation doses based on test results to prevent health issues arising from long-term high or low intake.

## 5 Conclusion

In conclusion, our study elucidates the causal relationship between trace elements, nutrients, and diabetes along with its complications through Mendelian randomization analysis. This insight offers a new perspective for subsequent mechanistic studies and provides new scientific evidence and strategies for the clinical application of micronutrients in the prevention and treatment of diabetes and its complications.

## Data availability statement

The datasets presented in this study can be found in online repositories. The names of the repository/repositories and accession number(s) can be found in the article/Supplementary material.

## Ethics statement

Ethical approval was not required for the studies involving humans because Ethical approval was not required for the study involving humans in accordance with the local legislation and institutional requirements. Written informed consent to participate in this study was not required from the participants or the participants’ legal guardians/next of kin in accordance with the national legislation and the institutional requirements. All data in this study were generated from studies approved by the respective ethics committees, and informed consent was granted to all subjects. Therefore, no additional approval from the institutional review board was required. The studies were conducted in accordance with the local legislation and institutional requirements. The participants provided their written informed consent to participate in this study. Written informed consent was obtained from the individual(s) for the publication of any potentially identifiable images or data included in this article.

## Author contributions

M-JJ: Writing – original draft, Writing – review & editing. LC: Writing – original draft, Writing – review & editing.

## Footnotes

^1^ https://gwas.mrcieu.ac.uk/

^2^ https://www.finngen.fi/en/access_results

^3^ https://r9.risteys.finngen.fi/

^4^ http://www.phenoscanner.medschl.cam.ac.uk/
